# Evaluation of Concordance Between Original Death Certifications and an Expert Panel Process in the Determination of Sudden Unexplained Death in Childhood

**DOI:** 10.1001/jamanetworkopen.2020.23262

**Published:** 2020-10-30

**Authors:** Laura Gould Crandall, Joyce H. Lee, Daniel Friedman, Kelly Lear, Katherine Maloney, J. Keith Pinckard, Peter Lin, Thomas Andrew, Kristin Roman, Kristen Landi, Heather Jarrell, Alex K. Williamson, J. C. Upshaw Downs, Kathy Pinneri, Christopher William, Joseph J. Maleszewski, R. Ross Reichard, Orrin Devinsky

**Affiliations:** 1 NYU Grossman School of Medicine, New York, New York; 2 Arapahoe County Coroner’s Office, Centennial, Colorado; 3 Erie County Medical Examiner's Office, Buffalo, New York; 4 University at Buffalo School of Medicine, Buffalo, New York; 5 Travis County Medical Examiner, Austin, Texas; 6 Mayo Clinic, Rochester, Minnesota; 7 White Mountain Forensic Consulting Services, Concord, New Hampshire; 8 University of New Mexico, Albuquerque; 9 Zucker School of Medicine at Hofstra/Northwell, Hempstead, New York; 10 Charleston Southern University, Charleston, South Carolina; 11 Montgomery County Forensic Services Department, Conroe, Texas

## Abstract

**Importance:**

The true incidence of sudden unexplained death in childhood (SUDC), already the fifth leading category of death among toddlers by current US Centers for Disease Control and Prevention estimates, is potentially veiled by the varied certification processes by medicolegal investigative offices across the United States.

**Objective:**

To evaluate the frequency of SUDC incidence, understand its epidemiology, and assess the consistency of death certification among medical examiner and coroner offices in the US death investigation system.

**Design, Setting, and Participants:**

In this case series, 2 of 13 forensic pathologists (FPs) conducted masked reviews of 100 cases enrolled in the SUDC Registry and Research Collaborative (SUDCRRC). Children who died aged 11 months to 18 years from 36 US states, Canada, and the United Kingdom had been posthumously enrolled in the SUDCRRC by family members from 2014 to 2017. Comprehensive data from medicolegal investigative offices, clinical offices, and family members were reviewed. Data analysis was conducted from December 2014 to June 2020.

**Main Outcomes and Measures:**

Certified cause of death (COD) characterized as explained (accidental or natural) or unexplained, as determined by SUDCRRC masked review process.

**Results:**

In this study of 100 cases of SUDC (mean [SD] age, 32.1 [31.8] months; 58 [58.0%] boys; 82 [82.0%] White children; 92 [92.0%] from the United States), the original pathologist certified 43 cases (43.0%) as explained COD and 57 (57.0%) as unexplained COD. The SUDCRRC review process led to the following certifications: 16 (16.0%) were explained, 7 (7.0%) were undetermined because of insufficient data, and 77 (77.0%) were unexplained. Experts disagreed with the original COD in 40 cases (40.0%). These data suggest that SUDC incidence is higher than the current Centers for Disease Control and Prevention estimate (ie, 392 deaths in 2018).

**Conclusions and Relevance:**

To our knowledge, this is the first comprehensive masked forensic pathology review process of sudden unexpected pediatric deaths, and it suggests that SUDC may often go unrecognized in US death investigations. Some unexpected pediatric deaths may be erroneously attributed to a natural or accidental COD, negatively affecting surveillance, research, public health funding, and medical care of surviving family members. To further address the challenges of accurate and consistent death certification in SUDC, future studies are warranted.

## Key Points

**Question:**

Does the US death investigation system underestimate the frequency of sudden unexplained death in childhood (SUDC)?

**Findings:**

In this case series of the SUDC Registry and Research Collaborative, 2 forensic pathologists from a pool of 13 independently reviewed 100 cases of SUDC. These reviewers were discordant with the original certifier’s cause of death opinion in 40% of cases, including 28 cases originally considered accidental or natural but adjudicated as unexplained by our review.

**Meaning:**

This study suggests that the US Centers for Disease Control and Prevention underestimates the rate of SUDC and that there is a low rate of consistency in death certification of sudden unexpected pediatric deaths.

## Teaser

This case series evaluates the frequency of sudden unexplained death in childhood (SUDC), explains its epidemiology, and assesses the consistency of death certification among medical examiner and coroner offices in the US death investigation system.

## Introduction

Sudden unexplained death in childhood (SUDC) is the unexpected death of a child aged 12 months or older for which no cause of death (COD) is identified after a thorough case investigation. In the United States in 2018, per *International Statistical Classification of Diseases, Tenth Revision, Clinical Modification *(*ICD-10-CM*) codes R96 to R99, SUDC was the cause of 392 deaths; 59% of which were among children aged 1 to 4 years, making SUDC the fifth leading category of death in this age group.^1^ The mean age of death is 25 months; most cases are boys (59%) found prone, having apparently died during sleep, with the peak incidence in winter months.^2^ Febrile seizures occurred in 28% of cases,^2^ a more than 10-fold greater rate than among the general population.^3^ African American children have a more than 2-fold risk of SUDC compared with non-Hispanic White, Asian, or Pacific Islander children.^2^

Research on SUDC is extremely limited. Unlike sudden unexplained infant death (SUID) and sudden infant death syndrome (SIDS), SUDC has received little public health or research support: we found fewer than 50 articles in the US National Library of Medicine, compared with more than 12 000 for SIDS. This more than 240:1 ratio contrasts with the 4:1 SIDS to SUDC death rates. While SIDS has declined by 50% during the past 20 years, SUDC rates among children aged 1 to 4 years doubled in Ireland (from 1994 to 2008; 0.8 to 1.8 deaths per 100 000 children) and remained stable in the United States (from 1999 to 2018; 1.3 to 1.4 deaths per 100 000 children).^1,4,5^

US medicolegal death investigations (MDI) are governed by state laws and administered primarily by medical examiner (ME) or coroner systems, with variations in investigation and certification practices.^6^ Unexpected pediatric deaths require investigations to determine the COD (eg, trauma, infection, cancer) and manner of death (MOD; ie, natural, accidental, homicide, suicide, undetermined). The COD is undetermined if the scene investigation, autopsy, and ancillary tests identify no COD. For children, the National Association of Medical Examiners (NAME) Panel on Unexpected Pediatric Deaths advocates classification as unexplained sudden death with or without intrinsic and extrinsic factors.^7^ Unlike sudden infant deaths in which a negative MDI is labeled SIDS or SUID, no diagnostic category has existed for deaths among children older than 1 year, which may bias some MEs and coroners to attribute unexplained deaths to nonlethal minor findings (eg, bronchitis) and certify them as explained deaths.^8^

We hypothesize that the US system underestimates SUDC frequency, which limits accurate death certification, research, and medical counseling for families. We assessed this hypothesis by subjecting a child death cohort to a systematic review of medical records, death scene investigation, autopsy findings, ancillary tests, and genetic data. A masked forensic pathologist (FP) panel reviewed all records. We sought to better define the epidemiology as well as the concordance of death certification.

## Methods

We reviewed the first 100 consecutively reviewed cases self-referred or referred by clinicians in the SUDC Registry and Research Collaborative (SUDCRRC) from 36 US states (92 cases [92.0%]), Canada (2 [2.0%]), and the United Kingdom (6 [6.0%]). The SUDCRRC, created at NYU Langone Health in 2014, connects academic investigators, FPs, and medical examiner and coroner partner offices to study sudden unexpected pediatric deaths. Each case enrolled had an autopsy and underwent a comprehensive masked review process. We included sudden unexpected deaths of children aged 11 months to 18 years in which the autopsy and investigation did not reveal a COD or if study investigators, clinicians, or parents deemed the COD unconfirmed by record review. For example, a child younger than 1 year was enrolled because of a sibling’s sudden death; our genetic studies identified an autosomal recessive (*PPA2*) disorder in both.^9^ Parental written consent was obtained, and the NYU institutional review board approved this study. Reporting is consistent with the the reporting guideline for case series.

We redacted decedent’s personal health information and other identifiers (eg, location of death, investigating office, professionals, COD, and MOD by original certifier) from medical records, investigative reports, photographs, ancillary studies, and histology slides (digitally scanned whole slide images available via password-protected online system). A pediatrician summarized the child’s medical records. Cardiovascular pathology and neuropathology consultants reviewed autopsy photographs, gross pathology findings, and histology slides and summarized their findings. Researchers conducted a detailed family interview and performed whole exome sequencing with clinically significant findings determined by consensus of 6 genetic experts from the Mayo Clinic, NYU, and Columbia.

For each case, 2 board-certified FP reviewers were masked by redacted records and each other’s review. To avoid possible bias from forensic training, the 2 reviewers had been trained at different academic centers. The entire study group included 13 board-certified FPs; 10 were NAME members, with 5 holding current or past leadership roles. After the case review, each FP completed a review form, offering their opinions of (1) whether there was sufficient information to determine COD, (2) whether they agreed with the original pathologist’s histology findings, (3) whether they agreed with the study’s cardiovascular pathology and neuropathology consultant reviews, (4) whether they identified cause(s) and manner of death, (5) the significant findings they identified, and (6) whether additional testing would clarify COD. The completed FP reviews and data were evaluated by an independent study co-investigator (L.G.C.). Discordant FP reviewer opinions on COD or MOD were adjudicated by a panel of at least 5 FP reviewers. The FP reviewers then received the genetic findings to assess whether the results affected their cause and manner of death opinions.

The original certifier and SUDCRRC causes and manners of death were compared. For deaths deemed unexplained by the SUDCRRC review, intrinsic and extrinsic factors were identified, per procedural NAME recommendations.^7^ Intrinsic factors include abnormal physiological or anatomic findings that may contribute to death but cannot explain the death or that represent natural conditions of unknown significance.^7^ Extrinsic factors are environmental factors that may threaten life but cannot explain the death given the overall investigative findings.^7^ Intrinsic and extrinsic factors do not convey a causal relation at this time. Deaths with insufficient information to assess for COD were considered undetermined due to insufficient data.

### Statistical Analysis

Results were analyzed between explained and unexplained deaths, concordant and discordant CODs, and the type of investigating office. Analyses were calculated using 2-tailed *t* tests for continuous variables and χ^2^ test of independence for categorical variables. The Fisher exact test was used in lieu of the χ^2^ test when the expected values were less than 5. *P* values were adjusted for type I errors using the Holm-Bonferroni correction for multiple comparisons and appear as adjusted *P* values.^10^ Odds ratios (ORs) with 95% CIs were calculated for significant associations between categorical variables. All statistical analyses were performed in Excel versions 16.34 to 16.37 (Microsoft Corp). Statistical significance was set at *P* < .05, and all tests were 2-tailed.

## Results

**Figure 1.**
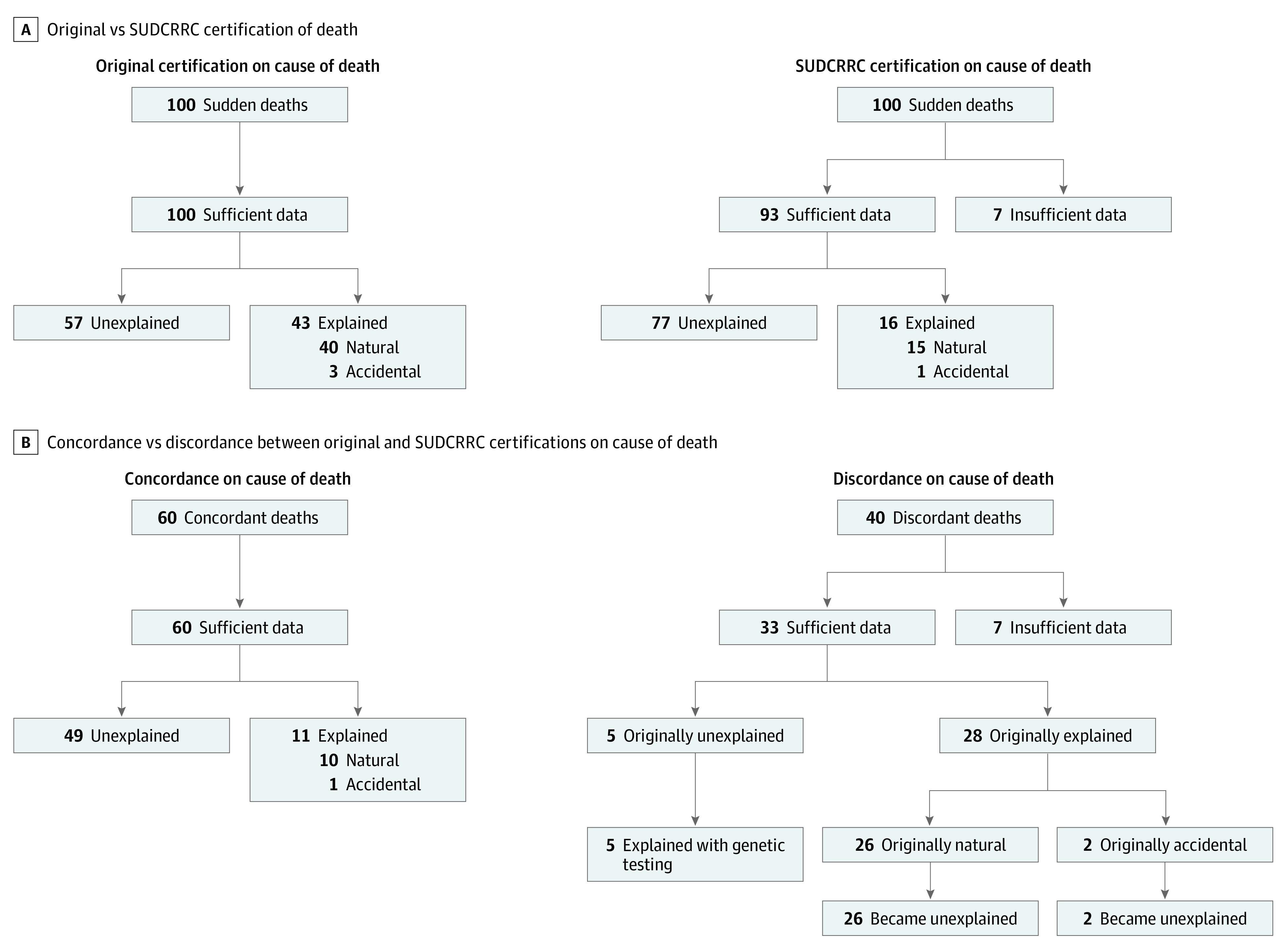
Original vs Sudden Unexplained Death in Childhood Registry and Research Collaborative (SUDCRRC) Certifications of Death

Of the 100 sudden unexpected child death cases reviewed, 58 (58.0%) were boys, with a mean (SD) age of 32.1 (31.8) months; 82 (82.0%) were White children, and 14 (14.0%) had mixed race, as described by family interview (Table 1). The original certifier classified 43 deaths (43.0%) as explained (ie, natural or accidental manners) and 57 (57.0%) as unexplained (Figure 1A). The original certifier considered 3 deaths (3.0%) accidental (2 [2.0%], accidental suffocation; 1 [1.0%], complication during general anesthesia). No deaths were considered suicide or homicide. The frequencies of various postmortem ancillary tests included microbiology (76 [76.0%]), vitreous electrolyte analysis (57 [57.0%]), toxicology (91 [91.0%]), metabolic disorder and disease testing (37 [37.0%]), genetic testing (3 [3.0%]), radiograph (58 [58.0]%), and computed tomography (CT) scans (2 [2.0%]). A death scene investigation was documented in 78 cases (78.0%). The original case certifiers were from ME offices (59 [59.0%]), coroner offices (35 [35.0%]), sheriff-coroner offices (4 [4.0%]), hospital autopsy services (1 [1.0%]), and private autopsy pathologists (1 [1.0%]).

**Table 1.** Case Summary Table

Factor	Cases, No./total No. (%)				*P* value^a^	Adjusted *P* value
	All (N = 100)	Explained (n = 16)	Unexplained sudden death (n = 77)	Undetermined due to insufficient data (n = 7)		
Boys	58/100 (58.0)	7/16 (43.8)	48/77 (52.3)	3/7 (42.9)	.17	>.99
Age, mo						
Mean	32.1 (31.8)	43.2 (44.7)	30.4 (29.6)	24.1 (13.2)	.29	>.99
Median (range)	22.1 (10.7-186.3)	21.4 (10.7-150.4)	23 (12.2-186.3)	16.5 (14.2-44.1)	NA	NA
Race/ethnicity^b^						
White	82/100 (82.0)	15/16 (93.8)	61/77 (79.2)	6/7 (85.7)	NA	NA
African American or Black	1/100 (1.0)	0/16	1/77 (1.3)	0/7		
Asian	3/100 (3.0)	0/16	2/77 (2.6)	1/7 (14.2)		
≥2	14/100 (14.0)	1/16 (6.3)	13/77 (16.9)	0/7		
Hispanic/Latino ethnicity	9/100 (9.0)	1/16 (6.3)	8/77 (10.4)	0/7		
With febrile seizure	39/100 (39.0)	6/16 (37.5)	33/77 (39.0)	3/7 (42.9)	.69	>.99

Abbreviation: NA, not applicable.

^a^ *P* values calculated between explained and unexplained deaths only.

^b^ *P* value was not calculable because expected values were less than 5 for χ^2^ test.

Our 2 masked FP reviewers were concordant in their COD opinion in 83 of 100 cases (83.0%). The remaining 17 cases were adjudicated by the FP panel as unexplained sudden death in 12 cases (70.6%) and explained sudden death in 5 cases (29.4%; 2 [40.0%], upper respiratory infections; 3 [60.0%], pathogenic genetic variants).

Overall, the SUDCRRC review process resulted in 16 explained cases (16.0%) (natural or accidental manners of death), 7 (7.0%) undetermined due to insufficient data, and 77 (77.0%) unexplained sudden deaths (Figure 1A, Table 2 and Table 3). We found that ME offices were more likely to have information considered sufficient to determine COD than a coroner office prior to correction of multiple comparisons (58 of 59 [98.3%] vs 29 of 35 [82.9%]; OR, 12.00; 95% CI, 1.38-104.41; *P* = .01; adjusted *P* = .14). Of 78 cases from ME or coroner offices adjudicated as unexplained sudden deaths or undetermined due to insufficient data, 18 (23.1%) had deficiencies in death scene investigation information. Autopsy and ancillary tests considered substandard included brain histology (22 [28.2%]), heart histology (16 [20.5%]), and microbiology or virology testing (19 [24.4%]). ME offices were 3.7-fold more likely to provide sufficient microbiology or virology testing than coroner offices prior to correction for multiple comparisons (43 of 51 [84.3%] vs 16 of 27 [59.3%]; OR, 3.70; 95% CI, 1.26-10.84, *P* = .01; adjusted *P* = .14).

**Table 2.** Explained Cases in Which COD Was Informed by Genetic Analysis Results

Patient No.	Sex	Age, mo	Original		SUDCRRC	
			COD	MOD	COD	MOD
10	Girl	20.8	Sudden unexplained death associated with atypical febrile seizures	Natural	Probable seizure disorder with pathogenic mutation associated with seizures	Natural
28	Boy	20.9	Sudden unexpected death in epilepsy	Natural	Sudden unexpected death in epilepsy with likely pathogenic mutation associated with seizures	Undetermined due to no scene investigation
37	Boy	39.9	Dilated cardiomyopathy	Natural	Hypertrophic cardiomyopathy associated with likely pathogenic variant associated with cardiomyopathy	Natural
45	Girl	47.9	Undetermined	Natural	Sudden unexpected death with likely pathogenic variant associated with cardiac channelopathy	Natural
46	Girl	135	Cardiac dysrhythmia of unknown etiology	Natural	Sudden cardiac arrhythmia with pathogenic variant associated with cardiac channelopathy	Natural
72	Girl	12.2	Unascertained	Natural	Cardiac arrhythmia or failure with likely pathogenic variants associated with sudden infantile cardiac failure^9^	Natural
99	Girl	26.1	Unable to definitively establish following investigation, autopsy, and laboratory evaluation	Undetermined	Probable seizure-related death associated with likely pathogenic variant associated with seizures	Undetermined due to no scene investigation
100	Girl	102	Undetermined	Undetermined	Sudden cardiac death with pathogenic variant associated with channelopathy	Natural
103	Girl	10.7	Sudden unexpected death in infancy	Undetermined	Cardiac arrhythmia or failure associated with likely pathogenic variants associated with sudden infantile cardiac failure^9^	Natural

Abbreviations: COD, cause of death; MOD, manner of death; SUDCRRC, Sudden Unexplained Death in Children Registry and Research Collaborative.

**Table 3.** Explained Cases in Which COD Was Not Informed By Genetic Analysis Results

Patient No.	Sex	Age, mo	Original		SUDCRRC	
			COD	MOD	COD	MOD
18	Boy	21.9	1a, Systemic inflammatory response syndrome; 1b, influenza A infection	Natural	Systemic inflammatory response syndrome; complications of influenza A viral infection	Natural
30	Boy	19.9	Acute bacterial infection secondary to primary viral (metapneumovirus) respiratory infection	Natural	Upper respiratory illness	Natural
31	Boy	16.7	Respiratory syncytial virus infection, pneumonitis, and probable *Staphylococcus aureus* sepsis.	Natural	Bronchiolitis of viral etiology	Natural
44	Girl	18.7	Rhinovirus and respiratory syncytial virus infection and other undetermined factors.	Undetermined	Respiratory syncytial virus laryngotracheobronchitis	Natural
64	Girl	33.7	Global hypoxic or ischemic encephalopathy due to prolonged resuscitated cardiopulmonary arrest due to complications of recurrent complex febrile seizures or epilepsy	Natural	Complications of febrile seizure	Natural
70	Boy	150	Sudden cardiac arrest during general anesthesia (with succinylcholine, propofol, and sevoflurane) for tonsillectomy	Accidental	Acute rhabdomyolysis with hyperkalemia and asystole associated with succinylcholine administration during tonsillectomy for chronic tonsillitis	Accidental
90	Boy	14.3	Myocarditis	Natural	Lymphocytic myocarditis	Natural

Abbreviations: COD, cause of death; MOD, manner of death; SUDCRRC, Sudden Unexplained Death in Children Registry and Research Collaborative.

There were no significant differences between the cases considered explained vs unexplained in regard to sex, age, race, ethnicity, or febrile seizure history. Whole exome sequencing significantly influenced the COD opinion in explained cases compared with implicating intrinsic factors in unexplained sudden deaths (9 of 16 [56.3%] vs 10 of 77 [13.0%]; OR, 8.61; 95% CI, 2.62-28.33; *P* < .001; adjusted *P* = .008). Of the 9 explained cases influenced by genetic results, 5 (55.6%) identified the COD in an unexplained case and 4 (44.4%) confirmed the original COD by pathological evidence (Table 2). Of the 7 explained cases not influenced by genetic results, 4 (57.1%) were respiratory illnesses (Table 3).

**Figure 2.**
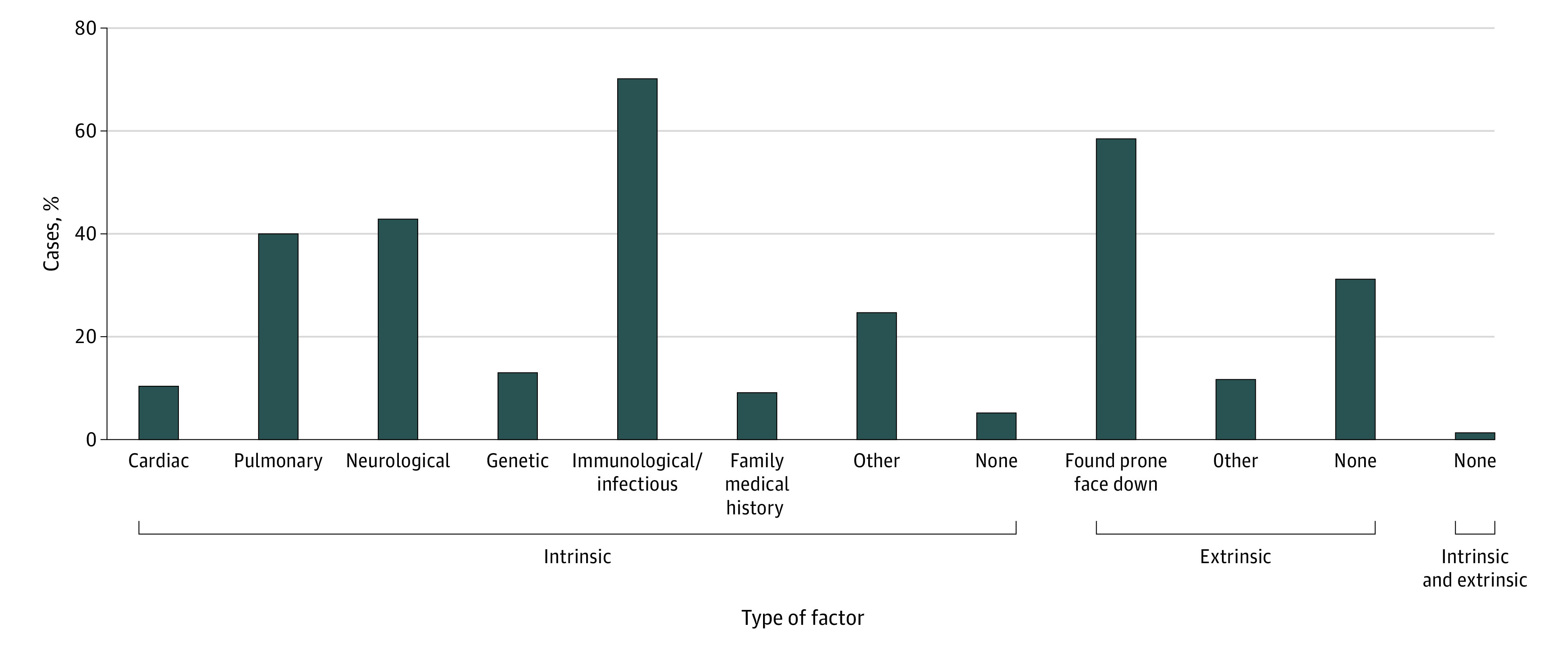
Percentages of 77 Unexplained Sudden Death Cases by Factor Type

The remaining 77 cases with sufficient information were determined unexplained sudden deaths with a mean (SD) 2.4 (1.5) intrinsic factors and 0.7 (0.5) extrinsic factors per case. Intrinsic factors included cardiovascular, pulmonary, neurologic, genetic, immunological or infectious, family medical history, other, or none. The most common types of intrinsic factors were immunological or infectious (54 [70.1%]), neurological (33 [42.9%]), and pulmonary (31 [40.3%]). The most common extrinsic factor was found prone or faced down (45 [58.4%]) (Figure 2). Only 1 case (1.3%) had no identified intrinsic or extrinsic factors.

### Concordance and Discordance with Original Certification

There was a 40% discordance (40 cases) in the COD determination between the original certifier and SUDCRRC (Figure 1). The SUDCRRC found 7 of 100 cases (7.0%) had inadequate information to assess COD, while original certifiers deemed them natural explained (4 [57.1%]) and undetermined (3 [42.9%]) deaths. Of the remaining 33 discordant cases, 5 (15.2%) were changed from unexplained to explained deaths by the SUDCRRC genetic analysis; 2 (6.1%), from accidental suffocation to unexplained sudden deaths; and 26 (78.8%), from natural explained deaths to unexplained deaths. The original explanation in these 26 cases were neurologic (11 [42.3%], with 9 [81.8%] with febrile seizure history), infection (7 [26.9%]), cardiac dysfunction (5 [19.2%]), and 1 (3.8%) each for severe hepatic steatosis, dehydration, and kidney dysfunction. Discounting the 7 cases considered undetermined due to insufficient data, the original certifier considered 54 deaths unexplained, while our review process yielded a 1.4-fold increase, with 77 unexplained deaths.

One or both FP reviewers partially disagreed with the original pathologist’s histological findings in 61 of 100 cases (61.0%). These disagreements affected COD in 16 cases (26.2%); the other 45 (73.8%) did not alter COD. Histology disagreements were most common for respiratory (29 [47.5%]), liver (12 [19.7%]), brain or spinal cord (6 [9.8%]), heart (7 [11.5%]), gastrointestinal tract (3 [4.9%]), and spleen (2 [3.3%]) tissues.

Our review process agreed with the original certifier in 11 explained deaths (10 [90.9%] natural, 1 [9.9%] accidental) and 49 unexplained deaths (Figure 1B). We found no significant differences among the cases that the SUDCRRC was discordant or concordant with the original certifier in regards to the size of jurisdiction, type of jurisdiction, type of office, type of MDI state system, and qualifications of the pathologist, coroner, or death scene investigator.

## Discussion

Our masked comprehensive review and adjudication process suggested that the SUDC rate may be higher than current US Centers for Disease Control and Prevention estimates (ie, 392 deaths in 2018), which would make SUDC the fourth leading category of death among children aged 1 to 4 years (231), after unintentional injuries (1226), congenital anomalies (384), and homicides (353) and more frequent than malignant neoplasms (326).^11^ SUDC is an extraordinarily underfunded and understudied problem given its devastating consequences. Our study findings disagreed with the original COD in 40% of cases, suggesting that for pediatric sudden deaths, the consistency of COD determination is lower than commonly assumed. However, concordance between our independent masked FPs was high, at 83%. Although in some cases there is no absolute correct answer, given that interpretation and judgement are involved, we sought a COD determination as accurate as possible and accept that certifications are medical opinions.

The quality of autopsy and death investigations varied greatly. While all cases were autopsied, only 3% had genetic testing performed, limiting accurate COD and preventative counseling to at-risk relatives of the decedents. A scene investigation, while critical—especially in sleep-related pediatric death investigations—was considered inadequate in nearly a quarter of cases. Two organs implicated in sudden death—the brain and the heart—each had inadequate examinations in more than 20% of cases. Compared with SUID investigations, SUDC investigations were less likely to include a death scene investigation, radiographs, metabolic testing, or genetic testing.^12^

Compared with the original certifier, we identified a higher frequency of unexplained cases. The original certifier often identified a natural cause (eg, bronchitis, pneumonia, or febrile seizure history) for which our review process did not consider the evidence sufficient for COD. We also identified frequent (ie, 61%) disagreement regarding histology findings, affecting COD opinion in 16% of cases.

Why do MEs and coroners more commonly classify deaths as explained when their peers in SUCDRRC consider them unexplained? For sudden and unexpected pediatric deaths, investigators may feel pressure to identify a COD. One mother, after reviewing the autopsy report, asked the ME why pneumonia was listed as the COD when her child had no fever and the pulmonary pathology was mild. He responded, “I had to put something down.”^7^ That certifier may have wanted to avoid the limbo of an unexplained certification that stokes fear regarding potential risk to relatives, suggests an incomplete investigation, or implies information was overlooked. SUDCRRC reviewers, who take a particular interest in the study of SUDC, may be more inclined to consider a death unexplained.

We identified omitted components of death investigation, including genetic, microbiology or virology, brain, and heart evaluations and thorough death scene investigations. However, discordance in opinion of COD was usually because of commission, ie, elevating what the SUDCRRC believed to be a minor pathological finding to COD. Our high rate of diagnostic ambiguity in death certification may also result from variation among investigating agencies’ policies and procedures, investigators’ viewpoints, resources, and pathologists’ experiences. To improve consistency in certification, the ambiguity and differences of opinion which are rarely recognized must be addressed.

To our knowledge, our study is the first to evaluate intrinsic and extrinsic factors in SUDC. Predominant immunological, pulmonary, and neurological intrinsic factors identify future research directions. We confirmed the predominant extrinsic factor was decedents found prone/faced down, consistent with functional impairment of reflexive autonomic, motor, and arousal responses, which in some cases may be because of the postictal state.^2,13,14^

Genetic (ie, whole exome sequencing) studies identified a likely COD in 9 of 93 cases (9.7%) and genetic variants as intrinsic factors in an additional 10 (10.8%). Future advances in understanding a more complete spectrum of pathogenic coding and noncoding variants will likely increase the yield from genetic studies. Moreover, enhanced genetic testing may allow for more accurate phenotypic spectra in diseases such as cardiomyopathies, for which subtle and currently overlooked findings may be significant and can guide testing.

The specificity of COD wording on death certificates determines accurate mortality surveillance using *ICD*-*10* coding. If a certifier lists undetermined in part 1 of the death certificate but adds a potential COD in part 2 (eg, febrile seizure history), that death will be coded as explained despite the certifier’s intentions.^7,15^ This affected 5 of our cases, affirming recommendations by NAME to include potential risk factors in the autopsy synoptic report and exclude them from the death certificate.^7^

The *ICD-10* codes for SUDC deaths (ie, R96-99) are not included in leading COD reports but qualify even with current underestimates.^16^ SUDC cases erroneously classified as natural explained deaths hamper a pediatrician’s ability to counsel family members. For example, pneumonia as the COD suggests no risk to surviving family members, but inherited disorders may affect family members (eg, siblings with cardiac disease due to autosomal recessive *PPA2 o*r epilepsy due to a pathogenic *SCN1A* variant inherited from an unaffected mosaic parent; Table 2).^9,17^

Our reviewers used CDC guidelines to classify cases as unknown if no COD was identified with a reasonable degree of medical certainty.^18^ Medical probability is a public health compromise that balances the need for epidemiological COD data, accurate data, and the scientific tenet to acknowledge uncertainty when evidence relies on judgement. Investigators with the same data reach different conclusions based on their location, training, and preferences.^19,20^

### Limitations

This study has limitations, including referral bias and retrospective accrual. However, our comprehensive and unbiased assessment of all data by expert FPs and geneticists reduced potential biases of local policies, pressures, or time constraints. Our independent expert reviews were followed by group adjudication for discordant cases. We found no other study with such a comprehensive masked forensic pathology review process.

Our cases were referred by certifiers or parents who felt additional investigation was warranted, biasing toward unexplained cases, although referring offices certified 43 cases (43.0%) as explained. For these cases, all data—from autopsy report wording to which anatomic regions and slides were selected—could bias to support the investigator’s COD theory. Annually in the United States, 21 000 infant and 12 000 child (ie, aged 1-18 years) deaths occur, and more than 3000 of these 33 000 deaths (9.1%) are certified as SUID or SUDC.^1,11^ Among our 43 originally explained cases, we found 4 had insufficient data and 28 were unexplained; only 11 cases were confirmed as explained in our review. The national overdiagnosis rate of explained cases is likely much lower than 74.5% (32 of 43). Yet, even a 15% overdiagnosis rate among 30 000 explained infant and child deaths yields approximately an additional 7500 SUID or SUDC deaths each year. We lack data to accurately extrapolate but posit that more than 5000 SUID or SUDC cases occur each year.

Without autopsy, COD errors in death certificates are common. Using solely clinical information, resident physicians incorrectly diagnose the COD in more than 75% of cases, while senior physicians err in 32% of cases.^21,22,23^ Even experienced FPs reviewing straightforward cases deemed not to require an autopsy were wrong in 28% of cases after autopsy revealed the COD.^24^ In these studies, cardiovascular causes were the most frequently overdiagnosed entities. Further, with identical case vignettes, US physicians more frequently certify a cardiovascular COD than colleagues in England or Sweden.^19^

## Conclusions

In this study, we found discordance in 40 of 100 cases of SUDC, suggesting that incidence is higher than current estimates. Underreporting of SUDC negatively affects public and professional awareness, research, public health funding, and medical care of family members. Real or perceived pressure to identify a COD may lead some to unwarranted determinations. Future research should address these challenges to provide more accurate COD certification in sudden pediatric deaths and explore potential causes for undetermined cases.
